# Problem-Solving Skills and Career Aspirations: The Role of Identity Acquisition and Self-Understanding in Italian Students

**DOI:** 10.3390/children13020285

**Published:** 2026-02-19

**Authors:** Emanuela Calandri, Enrico Vitolo, Jessica Verdiglione, Martina Bollo, Angelica Arace, Paola Ricchiardi, Teodora Lattanzi, Marianna Campione, Silvia Gattino

**Affiliations:** 1 Department of Psychology, University of Turin, Via Verdi 10, 10124 Turin, Italy; emanuela.calandri@unito.it (E.C.); jessica.verdiglione@unito.it (J.V.); martina.bollo@unito.it (M.B.); silvia.gattino@unito.it (S.G.); 2 Department of Philosophy and Education Sciences, University of Turin, Via Sant’Ottavio 20, 10124 Turin, Italy; angelica.arace@unito.it (A.A.); paola.ricchiardi@unito.it (P.R.); teodora.lattanzi@unito.it (T.L.); 3 Office for Teaching and Student Services, University of Turin, Via Sant’Ottavio 12/b, 10124 Turin, Italy; marianna.campione@unito.it

**Keywords:** adolescence, problem-solving, identity acquisition, self-understanding, educational programs, career aspirations, career decision-making

## Abstract

**Highlights:**

**What are the main findings?**

- Identity acquisition processes and self-understanding mediated the relationship between adolescents’ problem-solving abilities and aspiration for an ideal occupation.
- Multigroup Structural Equation Model analyses produced group-specific path estimates, indirect effects, and invariance tests.

**What are the implications of the main findings?**

- Educational interventions should foster problem-solving abilities from early developmental stages to support adolescents’ future-oriented goals and self-actualization.
- Vocational and educational programs should prioritize identity acquisition and self-clarity as core components of effective future-oriented guidance for adolescents.

**Abstract:**

**Background/Objectives:** Adolescence is a critical developmental period in which individuals are required to orient themselves toward the future and construct a coherent life plan, including educational and career aspirations. Future orientation is closely linked to identity development and self-understanding, which allow adolescents to integrate past, present, and anticipated future selves. Among the personal resources supporting this process, problem-solving skills play a key role by enabling effective coping with challenges and informed, goal-directed decision-making. This study examined the association between problem-solving skills and adolescents’ aspirations for an ideal occupation, and tested whether this relationship was mediated by identity acquisition and self-understanding, with attention to gender differences. **Methods:** A quantitative study design was adopted. Participants were 2443 Italian adolescents (aged 15–19 years) attending upper secondary schools. They completed self-report measures assessing perceived problem-solving skills, identity acquisition, self-understanding, and aspiration for an ideal occupation. Two multigroup mediation models were tested using structural equation modeling, examining identity acquisition and self-understanding as mediators and comparing pathways across genders. **Results:** Problem-solving skills were indirectly associated with stronger aspirations toward an ideal occupation through identity-related processes. Identity acquisition mediated this association only among females, whereas self-understanding emerged as a significant mediator for both females and males, with partial mediation among females and full mediation among males. **Conclusions:** Overall, although constrained by the cross-sectional design, the findings are consistent with the notion that problem-solving skills contribute to future-oriented career aspirations chiefly by promoting identity coherence and self-clarity. These findings highlight the importance of integrating problem-solving training with identity-focused interventions in educational and career guidance programs, while considering gender-specific developmental pathways.

## 1. Introduction

During adolescence, particularly from middle to late adolescence, young people are expected to participate in developing their life plan. By life plan, we mean the collection of desires, dreams about the future, and the actions necessary to achieve personal goals. Life plans influence adolescents’ current decisions, as the future is built day by day and the choices made will affect what lies ahead [1,2]. Building a life plan is one of the developmental tasks [3,4] that begins in adolescence, takes shape in young adulthood, and involves the ability to orient oneself towards the future. This ability forms the basis for constructing a life plan and is an important component of identity development. If we consider identity as a self-generated organization of personality that emerges from the integration of the self over time and within social contexts [5], with a focus on the temporal dimension, identity development can be seen as the integration of the past and present self with the future self [6]. Contemporary developmental research highlights that future orientation is not merely a cognitive anticipation of forthcoming events but a motivational and identity-based process that connects present actions with anticipated future selves [7,8]. This capacity forms a core component of identity development. If identity is conceptualized as a self-organizing and self-generated structure that emerges through the integration of personal experiences across time and social contexts [5], then identity development can be understood as the progressive integration of past, present, and anticipated future selves [2,9].

The ability to imagine a future self is therefore the culmination of a process of understanding time that begins at birth and progresses throughout childhood and adolescence [10,11]. The importance of the ability to orient oneself towards the future is supported by the expectancy–value theory, which postulates that individuals modify their current behaviour based on their assessment of future outcomes [12,13], specifically: how an outcome is evaluated [14] and the probability that the outcome will occur [15]. Future orientation can influence a person’s academic performance and health. It is an evolutionary process, and some research has shown that this process improves with age [16]. Empirical research indicates that future orientation plays a significant role in academic achievement, motivational engagement, and health-related behaviors. Adolescents with stronger future orientation demonstrate greater academic persistence, more adaptive goal-setting strategies, and healthier behavioral patterns [17,18]. From a developmental perspective, future orientation represents a progressive and evolving capacity. Research in developmental psychology suggests that this ability increases with age, particularly from middle to late adolescence, as cognitive control, abstract reasoning, and temporal integration mature [19,20,21].

Consistent with these findings, late adolescents typically report higher levels of future planning [22,23], stronger motivational investment in long-term goals, reduced endorsement of fatalistic beliefs, and a more coherent time perspective compared to younger adolescents [9,19]. These developmental changes reflect the increasing capacity to connectpresent actions with anticipated future selves, thereby supporting more structured and intentional life planning.

### 1.1. Career Aspirations

Among the various choices that characterise life planning is the choice of profession or career, which is one of the most important decisions made during adolescence. Career exploration is conceptualised as a lifelong process that begins in adolescence, enabling individuals to understand the world of work and become more self-aware [24]. Often, choosing a profession is a source of considerable anxiety and concern among young people; this choice is influenced by several key factors, including family pressure, social pressure, circumstances, and past achievements [25], as well as individual characteristics.

### 1.2. Identity Acquisition and Self-Understanding

Compared to the latter, career choice is closely related to the identity development of adolescents and emerging adults [26]. Identity can be seen as a self-portrait composed of multiple domains, one of which is the career path an individual wishes to pursue. According to Erikson [6], the fifth developmental stage, or crisis, is identity versus identity confusion, which occurs during adolescence. During this stage, adolescents are challenged to determine who they are, what they value, and where they are heading in life. As they begin to realise that they will soon be responsible for themselves and their lives, they try to figure out what their lives will be like [4,27]. In addition to acquiring identity, the development of adequate self-understanding and a good level of internal coherence also plays an important role in choosing a professional career.

Self-understanding is the individual’s cognitive representation of the self. An adolescent’s self-understanding is based on the various roles and categories that define who they are [28]. Although self-understanding provides the rational basis, it does not constitute the whole of personal identity [4]. Self-understanding refers to internal consistency and stability and is one of the key personal factors affecting the career decision-making process. During high school, adolescents typically develop greater self-understanding, which enables them to recognise their strengths and pursue personal goals. This is essential for making future-oriented decisions about education and career paths [29]. Therefore, before choosing a career, adolescents should consider the pros and cons of their options and conduct a self-analysis to ensure their aptitudes align with their chosen occupation. In this context, career guidance is essential in helping students make informed career decisions [30].

The maturation of self-knowledge is associated with a strengthened sense of coherence. Individuals with a stronger sense of coherence tend to have fewer dysfunctional career thoughts and are better able to manage the decision-making process. Those with a strong sense of coherence typically perceive stressors as challenges rather than burdens, believing they have the resources to meet life’s demands. Consequently, they are more likely to view the problems and difficulties associated with making career decisions as manageable and worthwhile [31].

### 1.3. Problem-Solving Skills

As mentioned, choosing a future career, and therefore the educational path to follow, is one of the main challenges of adolescent development. As indicated by the WHO [32], to effectively face significant challenges, individuals must develop adequate problem-solving skills. Throughout our lives, we encounter different types of problems. For an infant, the priority is to reach a toy that is difficult to access due to their stage of psychomotor development. A school-aged child may need to solve an arithmetic problem, a teenage student may have to tackle a complicated chemistry problem or resolve a conflict with aromantic partner. Young adults may need to achieve career goals or address issues with a long-term partner, while adults or senior citizens may have to deal with financial or health concerns. These are all examples of how problem-solving skills are relevant throughout the lifespan. Furthermore, these problems share a common element: an individual faces an obstacle and implements a strategy to overcome it and achieve their goal [33]. Problem-solving skills can be defined as a cognitive-emotional-behavioural process that includes effective ways of dealing with everyday problems and of defining and exploring individual or group efforts [34]. Solving a problem is synonymous with dealing with it. In fact, people evaluate problem-solving differently: some consider themselves capable of dealing with a problem, while others may believe they lack such abilities. This directly affects an individual’s performance in solving their problems [35]. Problem-solving skills develop during adolescence, and during this period, teenagers must face and resolve numerous problems. Furthermore, it has been highlighted that having well-developed problem-solving skills increases adolescents’ well-being [36].

### 1.4. Aims and Hypotheses

Problem-solving skills can therefore be an essential resource for adolescents when choosing a professional career, as these skills also contribute to the development of identity and the maturation of adequate and coherent self-understanding. Therefore, our study aimed to investigate, in a sample of Italian adolescents, the links between problem-solving skills and the ability to orient oneself towards the future, here considered as aspiration for an ideal occupation. We hypothesise that adolescents with greater problem-solving skills are also better able to define their personal aspirations for an ideal profession. We further hypothesise that problem-solving skills influence aspirations towards one’s ideal profession both directly and indirectly, through a more defined acquisition of identity and improved self-understanding and self-coherence. In particular, the aims were:

1. To investigate the mediating role of identity acquisition and self- understanding and self-coherence in the association between problem-solving skills and aspiration for an ideal occupation;
2. To examine whether these mediated relationships vary according to adolescents’ gender.

## 2. Materials and Methods

### 2.1. Procedure and Participants

Participants were recruited from forty-four high schools in northern Italy and included only students attending the third, fourth, and fifth years of upper secondary education. All participants provided informed consent, and their personal data were anonymized using alphanumeric coding. As the participants were underage, parental informed consent was also obtained, in accordance with current national and international laws and research guidelines. All participants reporting a history of mental health disorders, neurodevelopmental disorders, or intellectual disability were excluded from the study. The study complied with the ethical standards of the relevant institutional and national research committees and followed the principles of the 1964 Declaration of Helsinki and its later amendments. Moreover, it was approved by the Institutional Review Board of the University of Turin (protocol code: 030357; date of approval: 10 May 2025).

All participants completed an online battery of self-report questionnaires during school hours under the supervision of trained research personnel, and responses werecollected anonymously. The entire questionnaire took approximately 30 min to complete. No imputation procedures were used, as cases with missing demographic data or missing responses to any instrument items were excluded prior to analysis.

The final sample consisted of 2443 adolescents aged 15 to 19 years (mean age = 16.80 years; standard deviation = 0.90; 57.6% female). Table 1 shows demographic characteristics of the samples included in the subsequent analysis.

### 2.2. Measures

To examine the relationships among problem-solving skills, identity dimensions, and aspirations for an ideal occupation, the following instruments were administered: the Problem-Solving Inventory (PSI) [37,38], the Test for the Achievement of Developmental Tasks in Adolescence (TCS-A) [39], and the Career Decision-Making Profile questionnaire (CDMP) [40]. In addition, a brief sociodemographic section assessing gender, age, and grade attended was administered, followed by the measures described below.

#### 2.2.1. Problem Solving Inventory—PSI

Problem-solving skills were assessed using the Problem-Solving Inventory (PSI), Form B [37,38], a 35-item self-report measure capturing individuals’ perceptions of their problem-solving styles rather than objective problem-solving performance. Items are rated on a six-point Likert scale ranging from 0 (strongly agree) to 5 (strongly disagree), with higher scores indicating a greater tendency to perceive oneself as an ineffective problem solver. For the purposes of the present study, all items were reverse-scored prior to analysis so that higher scores reflected higher levels of perceived problem-solving ability. The PSI yields a total score and includes three factor-analytic dimensions: “Problem-Solving Confidence”, “Approach-Avoidance Style”, and “Personal Control” [41]. In the present study, only the PSI total score was used, which showed a Cronbach’s α of 0.78 (McDonald’s ω = 0.80). Although lower than previously reported values [38], this level of internal consistency is considered acceptable according to established guidelines [42,43].

#### 2.2.2. Test for the Achievement of Developmental Tasks in Adolescence—TCS-A

Achievements of developmental tasks in adolescence were assessed using the TCS-A [39], a multidimensional self-report instrument comprising 120 items rated on a four-point Likert scale. The TCS-A assesses adolescents’ perceived attainment of key developmental tasks across a broad range of domains central to normative developmental progression. The instrument consists of 12 scales, each reflecting a core developmental task related to puberty and sexuality, cognitive and relational competencies, and identity development. For the present study, only the “Identity Acquisition” and “Self-Knowledge and Self-Coherence” subscales were used. The original validation study reported satisfactory psychometric properties for both subscales, with Cronbach’s α = 0.87 [39]. In the present study, internal consistency was similarly adequate, with Cronbach’s α = 0.86 (McDonald’s ω = 0.87) for Identity Acquisition, and with Cronbach’s α = 0.87 (McDonald’s ω = 0.86) for Self-Knowledge and Self-Coherence. For clarity purposes, the Self-Knowledge and Self-Coherence subscale has been renamed Self-understanding.

#### 2.2.3. Career Decision-Making Profile—CDMP

The CDMP [40] consists of 36 items rated on a 7-point Likert scale ranging from 1 (strongly disagree) to 7 (strongly agree), assessing the main profiles adopted by individuals engaged in career decision-making processes, which may be related to both personality and situational influences. The dimensions assessed cover key aspects of the decision-making process, including information management, control and effort, decisional style and timing, interpersonal influences, and goal orientation (e.g., aspiration for an ideal occupation and willingness to compromise). The CDMP has previously been used in the Italian context, showing good psychometric properties (Cronbach’s α ranging from 0.63 to 0.81) when administered among adolescents [44]. For the present study, in accordance with our main aims, only the “Aspiration for an Ideal Occupation” scale was used, which showed a satisfactory internal consistency value of α = 0.87 (McDonald’s ω = 0.87).

### 2.3. Data Analysis

Data analyses were conducted in RStudio (version 2025.09.2+418) using the *lavaan* package (version 0.6-19) [45]. We tested two theoretically parallel multigroup mediation models that differed only in the identity-related mediator. In both models, Problem Solving (PSI total score) was specified as the predictor and Aspiration for an Ideal Occupation (CDMP subscale) as the outcome. Identity Acquisition (TSC-A subscale) was specified as the mediator in Model 1, whereas Self-understanding (TSC-A) served as the mediator in Model 2.

Each model was estimated using a multigroup structural equation modeling (SEM) framework by gender. Models were estimated using maximum likelihood (ML), which is appropriate for large samples and provides reliable standard errors and confidence intervals for indirect effects [46,47]. Bias-corrected bootstrap resampling (2000 draws) was implemented specifically to derive confidence intervals for the indirect effects, as recommended for mediation analyses given the non-normal distribution of product terms [47,48].

For each mediation model, we first estimated a fully unconstrained (free) multigroup SEM in which the structural paths were allowed to vary across groups. Indirect effects were computed as the product of the path from Problem Solving to the mediator (a) and the path from the mediator to the outcome (b). Structural invariance was then examined through a series of nested models in which individual paths (a, b, and c′) were constrained to equality across groups and compared to the unconstrained model using chi-square difference tests (Δχ^2^) [49].

Group differences in indirect effects were formally evaluated using Wald tests, which directly compared indirect effects across groups and provided a robust assessment of moderated mediation [50,51]. Standardized parameter estimates and delta-method standard errors are reported to facilitate interpretation. Together, these analyses allowed us to assess both the presence of mediation within groups and the invariance of mediated pathways across gender. Interpretations of invariance were based on the combined results of the Δχ^2^ tests, Wald tests, and comparisons of standardized parameter estimates [52].

## 3. Results

Table 2 shows descriptive statistics of the variables included in the analyses, namely the PSI total score, Identity Acquisition and Self-understanding (TCS-A subscales), and Aspiration for an Ideal Occupation (CDMP subscale). Skewness and kurtosis values for all dimensions were below 2 and 7, respectively, indicating no substantial departures from normality; therefore, no data transformations were required [53].

Table 3 presents descriptive statistics of the selected variables for male and female subsamples, along with the results of independent-samples t-tests comparing the two groups. Significant differences between males and females were found for all variables, with males scoring higher than females across all dimensions. However, differences were considered small, as indicated by Cohen’s *d* below 0.30 [54].

Before testing the mediation models, bivariate correlations among the selected variables were examined. Significant correlations were found across all dimensions. The strongest association was between TCS-A Identity Acquisition and CDMP Aspiration for an Ideal Occupation dimensions (*r* = 0.484; *p* < 0.001). Table 4 summarizes these results.

### 3.1. Model 1: Problem Solving → Identity Acquisition → Aspiration for an Ideal Occupation

Model 1 examined whether Identity Acquisition mediated the association between Problem Solving and Aspiration for an Ideal Occupation, and whether this process differed by gender. Overall, the results revealed a gender-specific pattern of mediation.

Among females, Problem Solving was indirectly associated with Aspiration for an Ideal Occupation through Identity Acquisition (Std. β = 0.060, *p* < 0.001, 95% CI [0.013, 0.034]). Higher perceived problem-solving abilities were associated with higher levels of identity acquisition (Path a: Std. β = 0.121, *p* < 0.001), which in turn were related to stronger aspirations towards an ideal occupation (Path b: Std. β = 0.496, *p* < 0.001). When Identity Acquisition was taken into account, the direct association between Problem Solving and Aspiration for an Ideal Occupation (path c′) was no longer significant (Std. β = −0.023, *p* = 0.329), suggesting a fully mediated effect. Among males, Problem Solving was unrelated to Identity Acquisition (Path a: Std. β = 0.056, *p* = 0.069), and no mediation effect was found (Std. β = 0.027, *p* = 0.070, 95% CI [−0.001, 0.021]).

**Figure 1:**
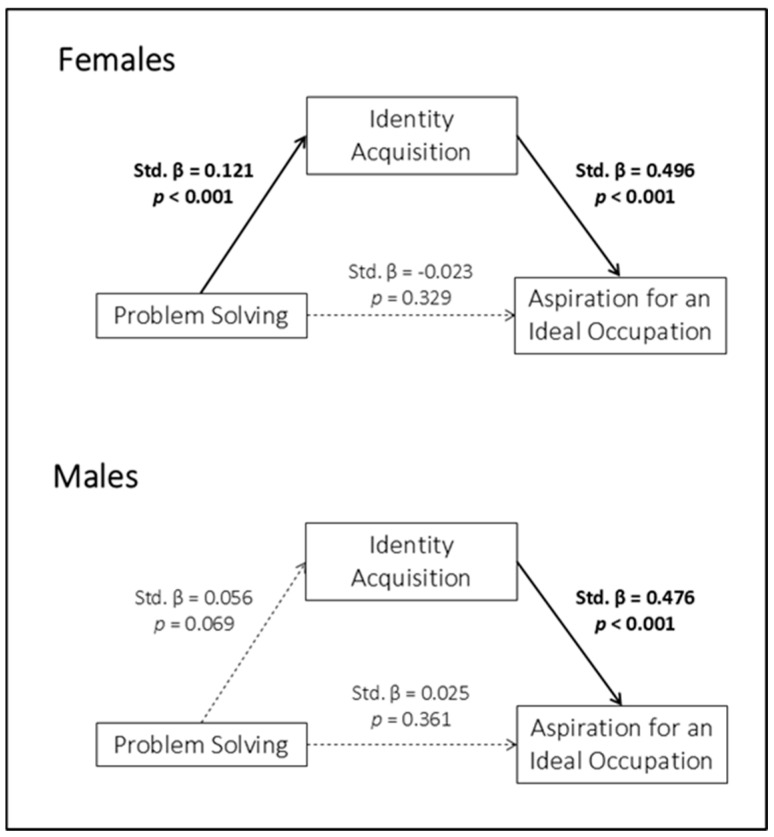
Mediation models (Model 1) among males and females.

Figure 1 presents a graphical representation of the mediation model for females and males.

To assess structural invariance across gender, equality constraints were imposed sequentially on each structural path. Constraining path a (from Problem Solving to Identity Acquisition) to equality across females and males did not result in a significant deterioration of model fit (Δχ^2^ = 3.39, *p* = 0.065), indicating invariance of this path. Similarly, constraining path b (from Identity Acquisition to Aspiration for an Ideal Occupation) and path c′ (the direct effect of Problem Solving on Aspiration for an Ideal Occupation) did not produce significant chi-square differences (Path b: Δχ^2^ = 0.24, *p* = 0.623; Path c′: Δχ^2^ = 1.78, *p* = 0.182), supporting equality of these parameters across gender.

**Figure 2:**
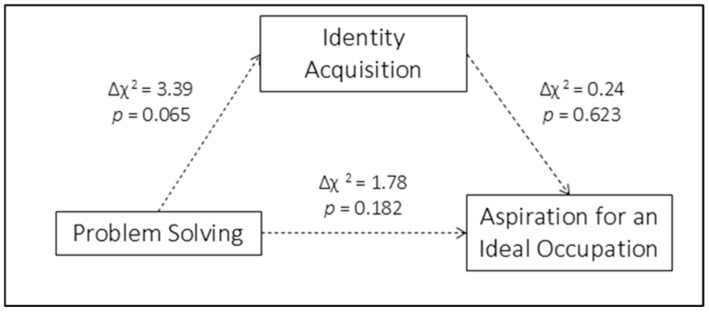
Difference in mediation models (Model 1) between males and females.

Consistently, the Wald test comparing indirect effects between gender groups was not significant (Wald = 3.60, *p* = 0.058), indicating that the mediated effect was statistically equivalent across gender, despite differences in the significance levels of indirect effects within-groups. Figure 2 provides a graphical representation of these results.

### 3.2. Model 2: Problem Solving → Self-Understanding → Aspiration for an Ideal Occupation

A second multi-group mediation model examined whether *Self-understanding* mediated the association between *Problem Solving* and *Aspiration for an Ideal Occupation*, and whether this mediation differed by gender. In contrast to Model 1, significant mediation effects were found in both gender groups, although with different patterns.

Among females, Problem Solving was positively associated with Self-understanding (path a: Std. β = 0.285, *p* < 0.001), which was in turn positively associated with Aspiration for an Ideal Occupation (path b: Std. β = 0.313, *p* < 0.001). The indirect effect was statistically significant (Std. β = 0.089, *p* < 0.001, 95% CI [0.027, 0.046]). When Self-understanding was included in the model, the direct effect of Problem Solving on Aspiration for an Ideal Occupation remained statistically significant (c′ path: Std. β = −0.052, *p* < 0.05), indicating a partially mediated association.

By contrast, among males, Problem Solving was positively associated with Self-understanding (path a: Std. β = 0.126, *p* < 0.001), which was in turn associated with Aspiration for an Ideal Occupation (path b: Std. β = 0.280, *p* < 0.001). The indirect effect was statistically significant (Std. β = 0.035, *p* < 0.001, 95% CI [0.006, 0.022]), whereas the direct effect of Problem Solving on Aspiration for an Ideal Occupation was no longer significant once the mediator was included in the model (path c′: Std. β = 0.016, *p* = 0.539). This pattern indicates that, among males, the association between Problem Solving and Aspiration for an Ideal Occupation was fully mediated by Self-understanding.

**Figure 3:**
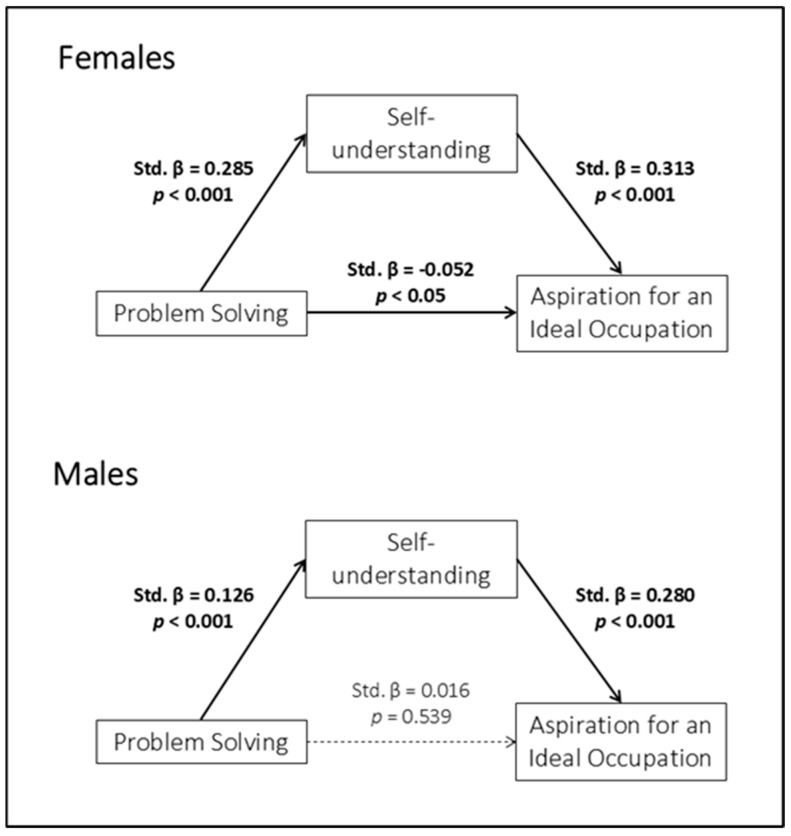
Mediation models (Model 2) among males and females.

Figure 3 presents a graphical representation of the mediation model for males and females.

**Figure 4:**
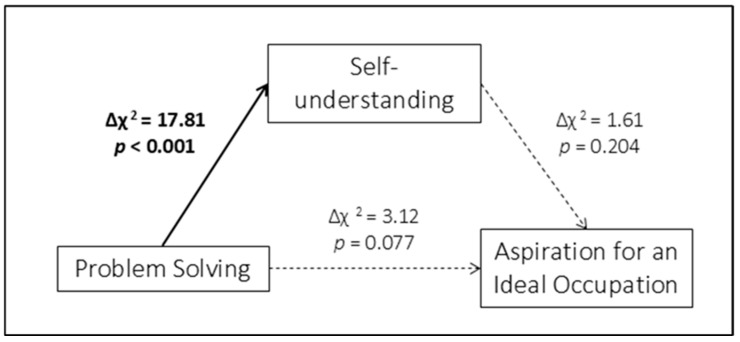
Difference in mediation models (Model 2) between males and females.

Nested model comparisons showed that imposing equality constraints on path a (from Problem Solving to Self-understanding) significantly worsened model fit across gender (Δχ^2^ = 17.81, *p* < 0.001), indicating that this path differed between males and females. In contrast, constraining path b (from Self-understanding to Aspiration for an Ideal Occupation) and path c′ (direct path from Problem Solving to Aspiration for an Ideal Occupation) did not result in significant chi-square differences (Path b: Δχ^2^ = 1.61, *p* = 0.204; Path c′: Δχ^2^ = 3.12, *p* = 0.077). Consistently, the Wald test assessing indirect-effect invariance was significant between genders (Wald = 17.13, *p* < 0.001), indicating that the mediated effect differed significantly across genders and therefore was not invariant between males and females. Figure 4 provides a graphical representation of these results.

### 3.3. Summary Across Models

Across both models, multigroup SEM analyses yielded gender-specific estimates of direct and indirect effects, as well as formal tests of structural invariance. In Model 1, a significant indirect effect emerged for females but not for males. Equality constraints imposed on paths *a*, *b*, and *c′* did not result in significant χ^2^ differences, and Wald tests were non-significant, indicating invariance of the indirect effect across gender.

In Model 2, significant indirect effects were observed in both gender groups; however, the association between Problem Solving and Aspiration for an Ideal Occupation was fully accounted for by Self-understanding only among males. In contrast to Model 1, constraining path a resulted in significant χ^2^ differences, and Wald tests indicated non-invariance of the indirect effect across gender. Table 5 provides a comparative summary of the indirect effects observed across models.

## 4. Discussion

This study examined the association between problem-solving skills and the ability to orient oneself towards the future, here understood as aspiration for an ideal occupation, and investigated whether this association was mediated by identity acquisition and self-understanding. In this paper, we have chosen to evaluate a model that uses problem solving as a predictor because it is a life skill that, if well mastered, can significantly improve adolescents’ career choices. Our results showed that problem-solving skills were indirectly associated with a greater ability to orient oneself towards the future through the mediation of identity acquisition and self-understanding, with gender differences. Specifically, the results indicate that the association between problem-solving skills and adolescents’ aspirations for an ideal occupation is not direct, but operates primarily through identity-related processes. Problem-solving skills appear to be relevant for career aspirations only insofar as they contribute to identity acquisition.

The first model revealed a gender-specific pattern. Among females, problem-solving skills were indirectly associated with aspiration for an ideal occupation through identity acquisition. Once identity acquisition was taken into account, the direct association between problem-solving and aspiration was no longer significant, suggesting that this relationship was explained by identity-related processes. Among males, no mediation effect emerged: problem-solving skills were unrelated to identity acquisition and to aspiration for an ideal occupation, although identity acquisition itself was positively associated with aspiration. These findings are consistent with models of identity formation and career development that emphasize identity acquisition as a key mechanism through which individual competencies are translated into career-related aspirations [55,56,57]. Among females, problem-solving skills were indirectly associated with aspiration for an ideal occupation through identity acquisition, suggesting that problem-solving skills can help define personal future career ambitions, especially when these skills influence identity formation processes. This finding aligns with previous evidence indicating that vocational identity mediates the relationship between decision-making resources and career outcomes, thereby explaining indirect rather than direct effects [58,59,60]. By contrast, among males, no mediation effect emerged: problem-solving skills were unrelated to both identity acquisition and aspiration for an ideal occupation, although identity acquisition itself was positively associated with aspiration. This pattern suggests that, while identity acquisition represents a relevant predictor of occupational aspirations for both genders, the processes through which cognitive skills contribute to identity development and subsequent aspirations may differ between males and females, implying that identity definition processes may be shaped by gendered developmental experiences [61]. Together, these findings highlight the central role of identity-related processes in shaping career aspirations, while also pointing to gender-specific pathways linking cognitive competencies and career aspirations.

The second model provided a somewhat different picture. Self-understanding mediated the association between problem-solving skills and aspiration for an ideal occupation in both females and males, albeit in different ways. Among females, this mediation was partial, as problem-solving skills retained a direct association with aspiration even after accounting for self-understanding. Among males, by contrast, the association between problem-solving and aspiration was fully explained by the indirect pathway through self-understanding. According to the “Career Construction Theory”, identity formation processes serve as a cognitive framework through which individuals integrate personal skills, beliefs, and experiences into career goals [62]. Indeed, it has been suggested that self-related constructs can act as mediating mechanisms between individual competencies and career outcomes [58]. Empirical evidence further indicates that vocational self-concept or related constructs can mediate relationships between antecedent variables and career decision effectiveness, highlighting the role of self-reflection and self-understanding in translating underlying competencies, such as possessed skills, into aspirational outcomes [63,64].

Taken together, our results support the conclusion that problem-solving skills contribute to adolescents’ orientation towards the future only when they are integrated into processes of self-construction, self-understanding, and coherence. Moreover, the results highlight that the mechanisms linking personal competencies to career aspirations follow partially different trajectories for males and females, underscoring the central role of identity-related dimensions in the development of vocational aspirations during adolescence [65]. Although our theoretical model conceptualized problem-solving skills as antecedents of career aspirations mediated by identity acquisition and self-understanding, it is important to acknowledge that developmental relationships in career-related constructs may be reciprocal rather than strictly unidirectional. Longitudinal researches have demonstrated reciprocal associations between career adaptability and vocational identity over time, indicating that individual resources and identity processes can influence each other in both directions during adolescence [66,67,68]. Similarly, other longitudinal evidence highlighted bidirectional dynamics between factors such as career exploration and subsequent aspiration trajectories, suggesting that developmental processes in career progression are dynamically interwoven [69]. Consequently, adolescents with stronger aspirations toward an ideal occupation may be motivated to further develop their problem-solving skills as part of their pursuit of career goals. Given the cross-sectional nature of the present study, causal inferences cannot be established, and future longitudinal research will be needed to examine reciprocal and dynamic relationships among these constructs.

Through his study, we highlighted the role of problem-solving skills in relation to the ability to define an important educational goal, such as identifying a profession considered ideal for oneself. Adolescents aged between 15 and 19 have opportunities to develop problem-solving skills during their school careers, and our findings suggest that these skills help clarify personal educational pathways, particularly among females. Furthermore, problem-solving skills appear to promote a clearer definition of educational identity among girls. This identity clarity, in turn, facilitates the identification of an educational goal—namely, an ideal profession—in both males and females. Acquiring adult identity is a long process, central to adolescence and continuing into young adulthood [70,71]. The finding that identity acquisition promotes clearer aspirations for future employment in both adolescent males and females challenges Erikson’s classic view of gender differences in identity development, according to which males were considered more career-oriented and females more family-oriented [6]. In line with previous studies on the development of identity in adolescence [61], our results show that the process of identity acquisition is associated with the development of ideal career expectations for both males and females. However, only among females was identity acquisition facilitated by problem-solving skills. This finding may indicate that the process of self-definition is more demanding for females than for males, which may relate to the need for females to counter persistent gender stereotypes, still strongly rooted in many cultures, such as the Italian one, especially with regard to professional fulfilment.

The process of identity acquisition is accompanied by the maturation of self-understanding, which increases significantly during adolescence as a result of the development of formal thinking. For example, adolescents are more likely than children to recognise that they possess multiple self-characteristics, depending on different contexts and specific roles assumed. The ability to reflect on their personal features enables youngpeople to begin outlining a training path aimed at self-fulfilment, particularly from a professional perspective. Confusion about oneself, however, makes it more difficult to understand what one wants to do in life and which educational path to pursue. The results of our study highlight the important role of problem-solving skills in adolescence that promote better and more consistent self-knowledge, which in turn facilitates the definition of personal expectations for the future, for both males and females.

The process of identity acquisition is accompanied by the maturation of self-understanding, which increases significantly during adolescence thanks to the development of formal thinking; for example, adolescents are more likely than children to understand that they possess several self-characteristics, also based on different contexts and specific roles assumed [27,28,72]. The ability to reflect on their personal features allows young people to begin to outline a training path aimed at self-fulfilment, especially from a professional point of view. Confusion about oneself, on the other hand, makes it more difficult to understand what one wants to do in life and also which educational path to take. The results of our study highlight the salient role in adolescence of problem-solving skills that promote better and more consistent self-knowledge, which in turn facilitates the definition of personal expectations for the future, for both males and females.

## 5. Conclusions

In conclusion, this study focused on a crucial period of life, widely regarded as one of the most delicate and challenging transitional periods in the life cycle. Between the ages of 15 and 19, expectations for the future become more central to development. From an applied perspective, this work highlights the importance of promoting problem-solving skills in adolescents to support their overall self-actualisation. Educational efforts to enhance problem-solving can be targeted specifically during adolescence, but should also be fostered in the family and school environment from early childhood. In this way, adolescents can be better equipped to face one of the most significant challenges of their age—a developmental task considered more critical for today’s adolescents than for those in the past—namely, defining future professional expectations and preparing for them through current educational pathways. In this process, the role of identity acquisition and self-clarity must be considered central, as these are essential aspects of any future-oriented programme aimed at adolescents.

### Limitations and Future Directions

Some limitations should be acknowledged. First, although the present study included students from 44 high schools, school membership was not available at the individual level in the final dataset. Consequently, it was not possible to adjust standard errors for clustering or to estimate multilevel models. While the large number of schools may reduce the magnitude of clustering bias, future studies should retain school identifiers and explicitly model school-level variance to strengthen the robustness of the findings. Second, the sample was slightly unbalanced with respect to gender, with a higher number of females than males. Although the distribution frequencies were relatively comparable, this imbalance may partially limit the generalizability of our findings. Third, the study was geographically restricted to northern Italy, thereby excluding a substantial portion of the national population. Future research should involve broader territorial samples to enhance the generalizability of the findings to the Italian context as a whole. Fourth, information about problem-solving skills was based on participants’ self-reports. Given the well-known limits of self-reported measures (e.g., social desirability, response biases, respondents’ lack of self-awareness, etc.) [73,74,75], future studies should consider integrating additional sources of information, such as teachers’ or parents’ reports. However, self-reported measures are largely adopted in psychological and personality research, given their statistical validity and accuracy in detecting subjective mental states and emotional processes [76,77,78]. Last, a longitudinal design over a longer period could allow investigation of developmental trajectories and causal links between the variables considered.

Despite these limitations, the instruments employed for data collection exhibited adequate levels of internal consistency and reliability. This methodological robustnessstrengthens the validity of the study and enhances confidence in the interpretation of the reported findings, further highlighting the empirical validity of the study. Moreover, the inclusion of a large sample size (N > 2000) represents a notable strength of the present study, providing robust evidence and offering valuable insights for researchers and practitioners interested in developing future-oriented educational and guidance programs for school-aged adolescents.

## Figures and Tables

**Table 1:** Demographics of the samples.

Total Sample (*N* = 2443)		
***Age***		
Range	15–19	
Mean	16.80	
SD	0.90	
	
***Gender***		
Males	*n* = 1036 (42.4%)	
Females	*n* = 1407 (57.6%)	
***Year of education***		
Third year	*n* = 1308 (53.5%)	
Fourth year	*n* = 693 (28.4%)	
Fifth year	*n* = 442 (18.1%)	
	
	
**Gender-split sample**		
***Age***	**Males**	**Females**
Range	15–19	15–19
Mean	16.81	16.79
SD	0.91	0.89
		
***Year of education***		
Third year	*n* = 568 (54.8%)	*n* = 740 (52.6%)
Fourth year	*n* = 287 (27.7%)	*n* = 406 (28.9%)
Fifth year	*n* = 181 (17.5%)	*n* = 261 (18.5%)

*Note: SD = standard deviation.*

**Table 2:** Descriptive statistics of the selected variables for the total sample (*N* = 2443).

	Min	Max	M	SD	Skewness	Kurtosis
PSI Total score	12	114	54.26	10.72	0.08	1.11
TCS-A Identity Acquisition	10	40	28.28	5.96	−0.17	−0.31
TCS-A Self-understanding	10	40	29.17	6.40	−0.26	−0.60
CDMP Aspiration for an Ideal Occupation	24	67	51.03	9.48	−0.33	−0.44

*Note*: M = mean; SD = standard deviation.

**Table 3:** Descriptive statistics of the selected variables for gender-split samples.

	Females (*n* = 1407)						Males (*n* = 1036)							
	Min	Max	M	SD	Skewness	Kurtosis	Min	Max	M	SD	Skewness	Kurtosis	*t* (2441)	Cohen’s *d*
PSI Total score	11	91	52.90	10.49	−0.10	0.55	20	113	56.11	10.77	0.29	1.63	−7.39 ***	−0.30
TCS-A Identity Acquisition	10	40	28.02	6.17	−0.19	−0.36	10	40	28.63	5.63	−0.10	−0.33	−2.48 *	−0.10
TCS-A Self-understanding	10	40	28.67	6.43	−0.24	−0.58	10	40	29.85	6.29	−0.29	−0.64	−4.55 ***	−0.19
CDMP Aspiration for an Ideal Occupation	26	66	50.50	9.46	−0.29	−0.55	24	67	51.74	9.47	−0.39	−0.26	−3.20 **	−0.13

*Note:* M = mean; SD = standard deviation. * *p* < 0.05; ** *p* < 0.01; *** *p* < 0.001.

**Table 4:** Correlations between the selected variables.

	TCS-A Identity Acquisition	TCS-A Self-Understanding	CDMP Aspiration for an Ideal Occupation
PSI Total score	0.098 ***	0.228 ***	0.049 *
TCS-A Identity Acquisition	-	0.458 ***	0.484 ***
TCS-A Self-understanding	-	-	0.290 ***

*Note:* * *p* < 0.05; *** *p* < 0.001.

**Table 5:** Comparison of indirect effects across models.

Model	Mediator	Gender	Indirect Effect (Std. β)	*p*-Value
Model 1	Identity Acquisition	Females	0.060	<0.001
Model 1	Identity Acquisition	Males	0.027	0.070
Model 2	Self-understanding	Females	0.089	<0.001
Model 2	Self-understanding	Males	0.035	<0.001

## Data Availability

The data presented in this study are available on request from the corresponding author due to ethical restrictions related to the protection of participants’ privacy, in accordance with European privacy normative (rif. EU-GDPR 2016/679).

## Abbreviations

The following abbreviations are used in this manuscript:

WHO	World Health Organization
PSI	Problem Solving Inventory
TCS-A	Test for the Achievement of Developmental Tasks in Adolescence
CDMP	Career Decision-Making Profile questionnaire
SEM	structural equation modeling
ML	maximum likelihood
